# Association between *de novo* variants of nuclear-encoded mitochondrial-related genes and undiagnosed developmental disorder and autism

**DOI:** 10.1093/qjmed/hcad249

**Published:** 2023-11-01

**Authors:** T Luo, J Pan, Y Zhu, X Wang, K Li, G Zhao, B Li, Z Hu, K Xia, J Li

**Affiliations:** Center for Medical Genetics & Hunan Key Laboratory of Medical Genetics, School of Life Sciences, Central South University, Changsha, Hunan 410008, China; Department of Birth Health and Genetics, The Reproductive Hospital of Guangxi Zhuang Autonomous Region, Nanning 530022, China; Center for Medical Genetics & Hunan Key Laboratory of Medical Genetics, School of Life Sciences, Central South University, Changsha, Hunan 410008, China; Center for Medical Genetics & Hunan Key Laboratory of Medical Genetics, School of Life Sciences, Central South University, Changsha, Hunan 410008, China; Department of Obstetrics and Gynecology, The First Affiliated Hospital of Anhui Medical University, Hefei 230022, Anhui, China; 4National Clinical Research Center for Geriatric Disorders, Department of Geriatrics, Xiangya Hospital & Center for Medical Genetics, School of Life Sciences, Central South University, Changsha, Hunan 410008, China; Department of Neurology, Xiangya Hospital, Central South University, Changsha, Hunan 410008,China; Bioinformatics Center, Furong Laboratory & Xiangya Hospital, Central South University, Changsha, Hunan 410008, China; 4National Clinical Research Center for Geriatric Disorders, Department of Geriatrics, Xiangya Hospital & Center for Medical Genetics, School of Life Sciences, Central South University, Changsha, Hunan 410008, China; Department of Neurology, Xiangya Hospital, Central South University, Changsha, Hunan 410008,China; Bioinformatics Center, Furong Laboratory & Xiangya Hospital, Central South University, Changsha, Hunan 410008, China; Center for Medical Genetics & Hunan Key Laboratory of Medical Genetics, School of Life Sciences, Central South University, Changsha, Hunan 410008, China; Center for Medical Genetics & Hunan Key Laboratory of Medical Genetics, School of Life Sciences, Central South University, Changsha, Hunan 410008, China; MOE Key Lab of Rare Pediatric Diseases & School of Basic Medical Sciences, Hengyang Medical School, University of South China, Hengyang, Hunan 410008, China; 4National Clinical Research Center for Geriatric Disorders, Department of Geriatrics, Xiangya Hospital & Center for Medical Genetics, School of Life Sciences, Central South University, Changsha, Hunan 410008, China; Department of Neurology, Xiangya Hospital, Central South University, Changsha, Hunan 410008,China; Bioinformatics Center, Furong Laboratory & Xiangya Hospital, Central South University, Changsha, Hunan 410008, China

## Abstract

**Background:**

Evidence suggests that mitochondrial abnormalities increase the risk of two neurodevelopmental disorders: undiagnosed developmental disorder (UDD) and autism spectrum disorder (ASD). However, which nuclear-encoded mitochondrial-related genes (NEMGs) were associated with UDD–ASD is unclear.

**Aim:**

To explore the association between *de novo* variants (DNVs) of NEMGs and UDD–ASD.

**Design:**

Comprehensive analysis based on DNVs of NEMGs identified in patients (31 058 UDD probands and 10 318 ASD probands) and 4262 controls.

**Methods:**

By curating NEMGs and cataloging publicly published DNVs in NEMGs, we compared the frequency of DNVs in cases and controls. We also applied a TADA-*denovo* model to highlight disease-associated NEMGs and characterized them based on gene intolerance, functional networks and expression patterns.

**Results:**

Compared with levels in 4262 controls, an excess of protein-truncating variants and deleterious missense variants in 1421 cataloged NEMGs from 41 376 patients (31 058 UDD and 10 318 ASD probands) was observed. Overall, 3.23% of *de novo* deleterious missense variants and 3.20% of *de novo* protein-truncating variants contributed to 1.1% and 0.39% of UDD–ASD cases, respectively. We prioritized 130 disease-associated NEMGs and showed distinct expression patterns in the developing human brain. Disease-associated NEMGs expression was enriched in both excitatory and inhibitory neuronal lineages from the developing human cortex.

**Conclusions:**

Rare genetic alterations of disease-associated NEMGs may play a role in UDD–ASD development and lay the groundwork for a better understanding of the biology of UDD–ASD.

## Introduction

Undiagnosed developmental disorder (UDD) and autism spectrum disorder (ASD) have considerable overlap and encompass a diverse range of conditions characterized by impaired development of the central nervous system.^1^ Their etiology involves pathophysiologic abnormalities in gene-sensitive individuals, with mitochondrial dysfunction possibly affecting a significant subset of children with UDD–ASD.^2^^,^^3^ However, the driving mechanisms of UDD–ASD with mitochondrial dysfunction are still not fully understood. The pathophysiological conditions of mitochondrial dysfunction may be caused by defects in mitochondrial proteins encoded by NEMGs or by changes in mitochondrial import mechanisms.

A previous study has reported that polymorphisms in NEMGs, such as *SLC25A12* and *PARK2*, are associated with ASD.^4^ Furthermore, a heterozygous variant in *SCN2A* (+/p.R607*) was found to reduce synaptic activity, which in turn affects mitochondrial function and results in ATP and energy storage dysfunction.^5^ The NEMG *VARS2* encodes a mitochondrial aminoacyl-transfer RNA synthetase that catalyzes the attachment of valine to transfer RNA for mitochondrial translation. *VARS2* mutations are reported to cause combined oxidative phosphorylation (OXPHOS) deficiency and early-onset mitochondrial encephalopathies.^6^ More recently, Replogle *et al*.^7^ reported that knockdown of transcriptional regulation factor (*TEFM*) and RNA degradation (*PNPT1*) lead to significant changes in the mitochondrial genome. These findings suggest that interference with the cellular mitochondrial crosstalk process can lead to impaired mitochondrial ATP production, leading to or contributing to the development of the disease. Therefore, dysfunctional homeostasis of cell mitochondria may involve nucleus–mitochondria crosstalk caused by genetic variants.

Although some genetic NEMG variants have been described in patients with neurodevelopmental disorder and mitochondrial dysfunction, correlation studies have been inconclusive, likely due to clinical and genetic heterogeneity between study groups and small sample sizes.^8^ Advances in whole-exome and whole-genome sequencing of large cohorts have increased the recognition of NEMG mutations in the human genome. Meanwhile, *de novo* variants (DNVs) with relatively stronger pathogenic effects are well-established causes of ASD and UDD.^9^ Accordingly, whether the DNVs in NEMGs are associated with UDD–ASD pathology remains unclear.

**Figure 1.**
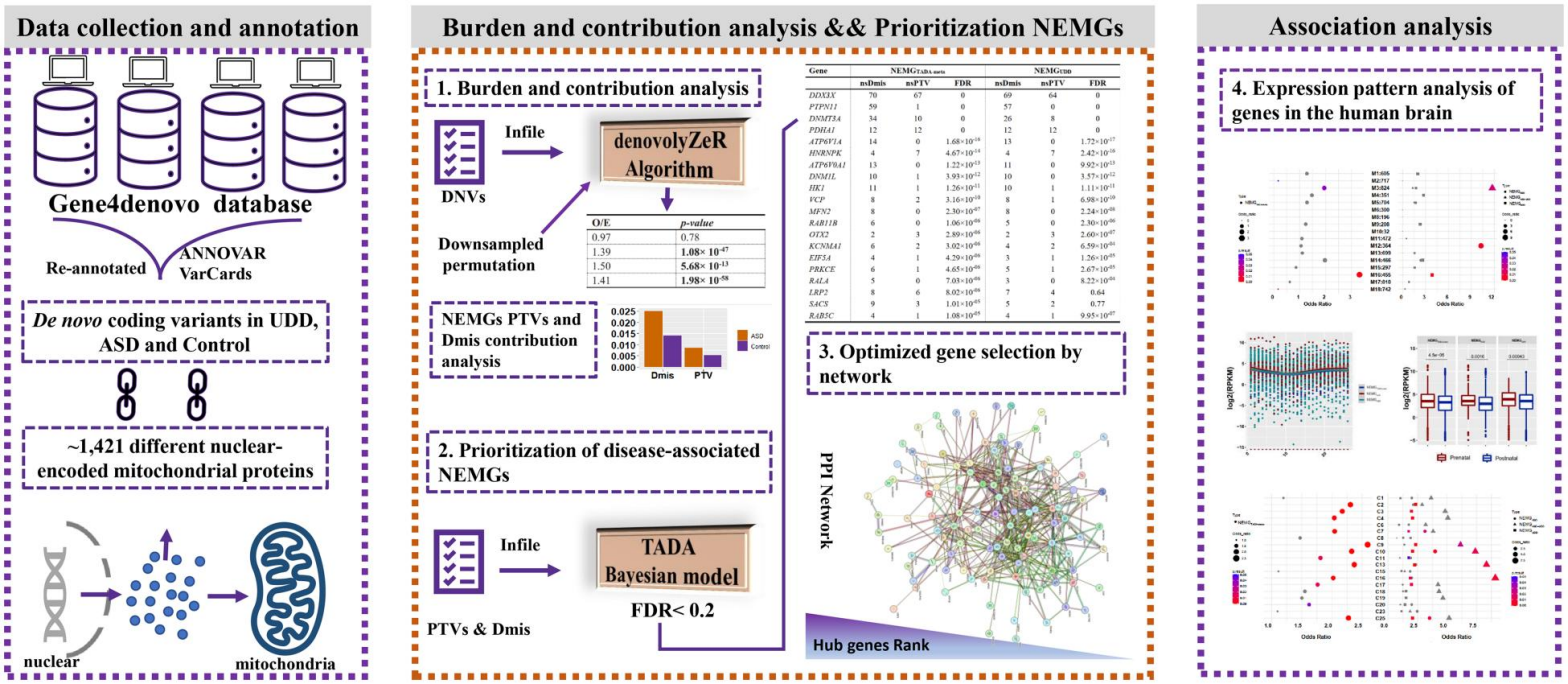
Flow chart of this study.

In this study, we compared coding DNVs in NEMGs between cases and controls and investigated the relationship between DNVs in NEMGs and genetically complex UDD–ASD. Furthermore, by performing integrative analysis, we prioritized potential disease-associated NEMGs that may contribute to elucidating the neurobiology of UDD–ASD (Figure 1). The study findings are expected to assist with patient stratification in clinical research and further aid in the development of more adequate treatment strategies for UDD–ASD.

## Methods

### Data collection and annotation

We manually curated NEMGs (Supplementary Table S1) from two mainstream mitochondrial databases: MitoCarta3.0^10^ and MitoMiner.^11^ MitoCarta 3.0, a comprehensive inventory consisting of 1136 human genes that encode proteins with detailed information on mitochondrial localization, sub-mitochondrial compartments and the assignment of genes to a customized ontology comprising many mitochondrial pathways (such as OXPHOS, mitochondrial apoptosis, mitochondrial autophagy and mitochondrial biosynthesis). We also collected a list of 1329 genes based on the ‘Integrated Mitochondrial Protein Index’ for known mitochondrial proteins available through MitoMiner (IMPI-2020-Q3pre). MitoMiner serves as a robust platform for investigating mitochondrial localization, providing an exceptional amalgamation of experimental sub-cellular localization datasets, tissue expression profiles, predictions of mitochondrial targeting sequences, comprehensive gene annotation and valuable links to phenotype and disease associations. Mitochondrial genes in these groups were excluded to form 1421 unique NEMGs (Supplementary Table S1).

All DNVs identified in patients with UDD–ASD and controls were downloaded from the Gene4Denovo database (http://www.genemed.tech/gene4denovo/home). The number of samples and DNVs from each cohort and other information on the cases and controls are listed in Supplementary Table S2. Only exonic DNVs in the NEMGs (Supplementary Table S3) were used for subsequent analysis. All variants were annotated with ANNOVAR.^12^ Protein-truncating variants (PTVs) consisting of frameshift, stop-gain/loss and canonical splice site disruptions and deleterious missense variants (Dmis) were considered ‘damaging DNVs’ (dDNVs).

### Burden and contribution analysis of PTVs and Dmis in NEMGs

The denovolyzeR^13^ tool was employed with default parameters to evaluate the enrichment for three variant classes (synonymous variant, missense variant and PTV), aiming to determine whether NEMGs exhibited a higher number of DNVs among patients with UDD–ASD compared to what would be expected by chance. This R package calculates the enrichment value by dividing the number of observed DNVs by the expected number of DNVs. Given that the distribution of DNVs follows a Poisson distribution, the expected number of DNVs for each variant class in the case/control groups was determined by the Poisson distribution and the population size. The *P*-values are calculated as the tail-probability of the expected distribution, derived either through Poisson approximations or simulations, under the null hypothesis of no association between each variant class and disease status. Compared with NEMGs, burden analysis of *de novo* PTVs and missense variants in five gene sets (Supplementary Table S4) were also evaluated in UDD–ASD, UDD and ASD. Given that the sample size of the case and control groups differed greatly, we performed 10 000 downsamplings to randomly select cases with the same control sample size; then, we used the permutation test to report the empirical *P*-values. In addition, we used ‘ascertainment differentials’,^14^ which are defined as the difference in the frequency of a specified variant type between the case and control groups, to estimate the contribution of NEMGs PTVs and Dmis to UDD–ASD patients.

### Prioritizing disease-associated NEMGs using a statistical model

Previous exome analyses have shown that even in unrelated individuals, the number of mutations carried in the same gene can provide considerable statistical power for the establishment of associations.^9^ Therefore, we used the transmitted and *de novo* association (TADA)^15^ test to prioritize genes associated with UDD–ASD. Under the null hypothesis, the TADA-*denovo* model generates random mutational data based on the background DNV rate of each gene of PTVs and Dmis and then calculates a *P*-values and a false discovery rate (FDR). The TADA-*denovo* model requires the following main parameters: the background DNV rate per gene of PTVs/Dmis and the number of disease risk genes of UDD–ASD, as described by He *et al*.^15^ for estimators. For each disease-associated NEMG, we used the loss-of-function observed/expected upper bound fraction (LOEUF) score and the missense constraint scores (mis_Z scores)^16^ to determine the intolerance to PTVs and Dmis, respectively. Low LOEUF scores at a cut-off value of 0.35 indicate greater intolerance to function in a gene. Mis_Z positive scores indicate more constraints, and *vice-versa*. A high *Z*-score indicates greater intolerance to missense variants.

### Hub genes identification

We visualized the PPI network using Cytoscape v3.7.2 (https://cytoscape.org/) and used the CytoHubba plug-in to identify the top 10 hub genes by 12 algorithms, including Degree, Edge Percolated Component, Maximum Neighborhood Component, Density of Maximum Neighborhood Component, Maximal Clique Centrality, Clustering Coefficient and six centralities (Bottleneck, EcCentricity, Closeness, Radiality, Betweenness and Stress).

### Permutation test

High-confidence UDD–ASD risk genes were extracted from Kaplanis *et al*.,^17^ Satterstrom *et al*.,^9^ Wilfert *et al*.^18^ and the SFARI database (genes with confidence levels of 1 and 2; https://www.sfari.org/), with a total of 627 genes being identified. We used PPI data from STRING to investigate whether the connections among the disease-associated NEMGs were statistically significant beyond random expectation for the 627 previously identified genes and three gene sets. PPI with a confidence score >600 in STRING was considered to be connected. The empirical *P*-values were calculated using 1 000 000 permutation tests.

### Gene sets

NEMGs were classified into five categories: (i) NEMG_TADA-meta_, comprising genes prioritized by the TADA-*denovo* model as dDNVs associated with UDD–ASD; (ii) NEMG_ASD_ and NEMG_UDD_, comprising NEMGs specific to ASD and UDD samples, respectively; and (iii) NEMG_ASD–UDD_, comprising overlapping genes within ASD and UDD.

### Expression pattern analysis of genes in the human brain

We used co-expression module data from Parikshak *et al*.^19^ to analyze the BrainSpan developmental RNA-seq data (www.brainspan.org). A total of 17 co-expression modules previously reported to be associated with the prenatal and postnatal cerebral cortex (excluding the M7 module, which is the gray module housing all non-co-expressed genes) were analyzed to determine whether each module was significantly enriched within each gene set (NEMG_ASD_, NEMG_UDD_, NEMG_ASD–UDD_ and NEMG_TADA-meta_) using Fisher’s exact test and *P-*values. Enrichment of all gene sets was used in R (R version 4.0.2):

fisher.test (matrix (c (x1,x2,x3,x4), alternative=¨two.sided¨)),

where *x1* is the number of each gene set expressed in the module, *x2* is the number of each gene set not expressed in the module, *x3* is the total number of genes expressed in the module minus *x1* and *x4* is the number of background genes minus *x1*, *x2* and *x3*. In addition, a Wilcoxon signed-rank test was performed to compare the expression distribution of NEMG_ASD_, NEMG_UDD_ and NEMG_TADA-meta_ between the prenatal and postnatal periods using the BrainSpan developmental RNA-seq data.

Gene ontology (GO) enrichment analysis was subsequently conducted using Metascape^20^ to understand the biological significance of the NEMG_ASD_ and NEMG_UDD_ gene sets. Only the terms with a minimum count of three, *P*_adjust_ value <0.05, and enrichment coefficient >1.5 were significant.

To evaluate the enrichment of specific cell types in the developing human cortex, Satterstrom *et al*.^9^ used data from Nowakowski *et al*.,^21^ which were divided into 25 cell-type clusters by *t*-distributed stochastic neighbor embedding analysis. Six cell-type clusters were excluded because they could not be associated with the cell types. To assess cell-type enrichment in the developing human cortex, we collated these re-analyzed results in subsequent analysis. We performed an enrichment analysis to determine whether each cell cluster of the developing human cerebral cortex was significantly enriched within each gene set (NEMG_ASD–UDD_, NEMG_ASD_, NEMG_UDD_ and NEMG_TADA-meta_).

## Results

### High DNV burden in NEMGs is associated with UDD–ASD

We curated 1421 NEMGs from two sources and excluded 13 genes encoded by mtDNA (Supplementary Table S1). We analyzed DNVs in the coding region of UDD–ASD cases (31 058 and 10 318 cases with the primary diagnosis of UDD and ASD, respectively) and 4262 controls and reannotated the information with the same pipeline as that used for our previous study.^22^

By comparing the proportion of individuals carrying DNVs in the UDD–ASD and control groups, we observed a statistically significant enrichment of PTVs and missense variants of NEMGs (PTV+missense, Observed/Expected [O/E] = 1.41, *P* = 1.68 × 10^−56^; PTV, O/E = 1.54, *P* = 1.39 × 10^−14^; missense, O/E = 1.38, *P* = 1.35 × 10^−44^). No enrichment in the UDD–ASD cohorts was observed regarding synonymous variants (O/E = 0.97, *P* = 0.78). No significant enrichment of any mutation category was identified in the controls (Table 1). Default parameters were used for denovolyzeR and Dmis was not assessed. A two-tailed Fisher’s exact test showed that Dmis were significantly enriched in the UDD–ASD cohort (odds ratio [OR] = 1.88, *P* = 0.00066). This burden of PTVs and missense variants persisted after dividing into UDD and ASD cohort, but UDD was more significant than ASD (Table 1). Compared to five reasonable sets of genes that may play a role in UDD_ASD, the NEMG variants exhibited the lowest enrichment of *de novo* PTV and missense variants (Supplementary Table S4). In addition, we performed 10 000 downsampled permutation tests to overcome bias resulting from the larger sample size for UDD–ASD than that for the controls (PTV, 0.0014 ≤ *P*_permutation _≤0.0090; missense, *P*_permutation _≤10^−3^; synonymous, 0.16 ≤ *P*_permutation _≤0.35; Supplementary Table S5). Further studies based on larger control sample sizes should be conducted to validate our conclusions. Overall, these findings suggest that the increased dDNV burden in NEMGs may contribute to UDD–ASD risk.

**Table 1.** Enrichment of DNVs in cases vs. controls in NEMGs by expectation analysis

	Observed	Expected	O/E	*P*-value
UDD–ASD (*N*=41 376)				
Synonymous	628	647.20	0.97	0.78
Missense	2057	1486.80	1.38	**1.35×10^−44^**
PTV	354	229.20	1.54	**1.39×10^−14^**
PTV+Missense	2411	1716.00	1.41	**1.68×10^−56^**
UDD (*N*=31 058)				
Synonymous	497	485.80	1.02	0.31
	1627	1116.00	1.46	**1.11×10^−46^**
PTV	286	172.10	1.66	**1.35×10^−15^**
PTV+Missense	1913	1288.10	1.49	**1.75×10^−59^**
ASD (*N*=10 318)				
Synonymous	131	161.40	0.81	0.99
Missense	430	370.80	1.16	**1.43×10^−3^**
PTV	68	57.20	1.19	0.088
PTV+Missense	498	427.90	1.16	**5.08×10^−4^**
Controls (*N*=4262)				
Synonymous	69	66.70	1.04	0.40
Missense	149	153.10	0.97	0.64
PTV	20	23.60	0.85	0.80
PTV+Missense	169	176.80	0.96	0.73

Autism spectrum disorder (ASD) and undiagnosed developmental disorder (UDD) are the most common neurodevelopmental disorders (NDDs). Syn, synonymous variants; mis, missense variants; PTVs, protein-truncating variants were defined as the composition of frameshift, stop-gain, stop-loss and canonical splice site disruption. We performed ‘denovolyzeR’ to estimate enrichment for four variants classes in NDDs vs. controls. O/E, observed/expected; Bold numbers indicate enrichment *P* < 0.05/(4 × 2).

### NEMGs dDNVs contribute to UDD–ASD risk in ∼1.49% of cases

Because NEMG dDNVs were significantly more frequent in patients with UDD–ASD than in controls, we estimated the percentage of these variants that were associated with UDD–ASD risk. The rate of PTV was 0.0047 in controls and 0.0086 in probands, yielding an ascertainment differential of 0.0039. Therefore, we estimated that ∼3.20% ([(0.0086 − 0.0047)/0.0086 × 1421]/20154) of PTV events in probands contributed to UDD–ASD risk in 0.39% of cases. For Dmis, the rate was 0.013 in controls and 0.024 in probands, with an ascertainment differential of 0.011. We estimated that only ∼3.23% ([(0.024 − 0.013)/0.024 × 1421]/20 154) of Dmis events in probands contributed to UDD–ASD risk in 1.1% of cases. In total, NEMGs dDNVs contributed to UDD–ASD risk in ∼1.49% of cases.

### Prioritization of disease-associated NEMGs

Using the TADA-*denovo* model, we prioritized 19 and 92 disease-associated NEMGs in the ASD and UDD groups, respectively. Given the aforementioned overlaps between ASD and UDD concerning NEMGs with dDNVs, integrated analysis confirmed 130 disease-associated NEMGs (Supplementary Table S6), including 24 candidate disease-associated NEMGs that did not reach the significance threshold (FDR≤0.2) in analyses of specific UDD–ASD subtypes. Of the 130 disease-associated NEMGs (Table 2), 62 had strong associations (FDR≤0.05), 32 had possible associations (0.05 < FDR≤0.1) and 36 had a suggestive association (0.1 < FDR≤0.2).

**Table 2.** The top 20 significant disease-associated NEMGs arising from the TADA-*denovo* model of prioritization

Gene	NEMG_TADA-meta_			NEMG_UDD_			NEMG_ASD_		
	nDmis	nPTV	FDR	nDmis	nPTV	FDR	nDmis	nPTV	FDR
*DDX3X*	70	67	<10^−16^	69	64	<10^−16^	1	3	1.49×10^−04^
*PTPN11*	59	1	<10^−16^	57	0	<10^−16^	2	1	8.97×10^−03^
*DNMT3A*	34	10	<10^−16^	26	8	<10^−16^	8	2	2.81 ×10^−10^
*PDHA1*	12	12	<10^−16^	12	12	<10^−16^	0	0	NA
*HNRNPK*	4	7	4.67×10^−14^	4	7	2.42×10^−16^	0	0	NA
*DNM1L*	10	1	3.93×10^−12^	10	0	3.57×10^−12^	0	1	0.39
*HK1*	11	1	1.26×10^−11^	10	1	1.11×10^−11^	1	0	0.63
*VCP*	8	2	3.16 × 10^−10^	8	1	6.98 ×10^−10^	0	1	0.41
*MFN2*	8	0	2.30×10^−07^	8	0	2.24 ×10^−08^	0	0	NA
*RAB11B*	6	0	1.06×10^−06^	5	0	2.30×10^−06^	1	0	0.50
*OTX2*	2	3	2.89×10^−06^	2	3	2.60×10^−07^	0	0	NA
*KCNMA1*	6	2	3.02×10^−06^	4	2	6.59×10^−04^	2	0	0.26
*EIF5A*	4	1	4.29×10^−06^	3	1	1.26×10^−05^	1	0	0.47
*PRKCE*	6	1	4.65×10^−06^	5	1	2.67×10^−05^	1	0	0.61
*RALA*	5	0	7.03×10^−06^	3	0	8.22×10^−04^	2	0	0.11
*LRP2*	8	6	8.02×10^−06^	7	4	0.64	1	2	0.099
*SACS*	9	3	1.01×10^−05^	5	2	0.77	4	1	0.0016
*RAB5C*	4	1	1.08×10^−05^	4	1	9.95×10^−07^	0	0	NA
*ETFB*	4	1	1.88×10^−05^	4	0	1.78×10^−04^	0	1	0.30
*POLG*	8	0	2.70×10^−05^	8	0	7.15×10^−06^	0	0	NA

nPTV, number of protein-truncating variants (PTVs) consists of frameshift, stop-gain, stop-loss and canonical splice site disruption in the case; nDmis, number of deleterious variations (Dmis) in the case; FDR, FDR from TADA-*denovo* analysis.

The intolerance gene often has a low LOEUF score and a greater mis_Z score; therefore, PTVs and Dmis occurring within constrained genes are of great interest.^23^ We found that 25 of the 130 disease-associated NEMGs had LOEUF scores <0.35 (Supplementary Table S7). These results implied that ∼19% of the 130 identified disease-associated NEMGs were intolerant to dDNVs. In addition, we found that 130 disease-associated NEMGs significantly connected with each of the unshared 627 UDD–ASD risk genes (Supplementary Figure S1) and three gene sets (genes encoded Fragile-X mental retardation protein targets, chromatin genes and genes encoded postsynaptic density proteins) based on the co-expression data from the human brain and PPI data compared with random expectation (Supplementary Figures S2–S4). Furthermore, we identified the top 10 hub genes (*PHB*, *ALDH18A1*, *MFN2*, *ATP5F1A*, *AKT1*, *ENO1*, *ECHS1*, *PDHA1*, *IDH2* and *ATP5F1B*) (Supplementary Table S8), which were revealed to be highly interconnected in a PPI network.

### Gene sets exhibit distinct expression patterns in the human brain

**Figure 2.**
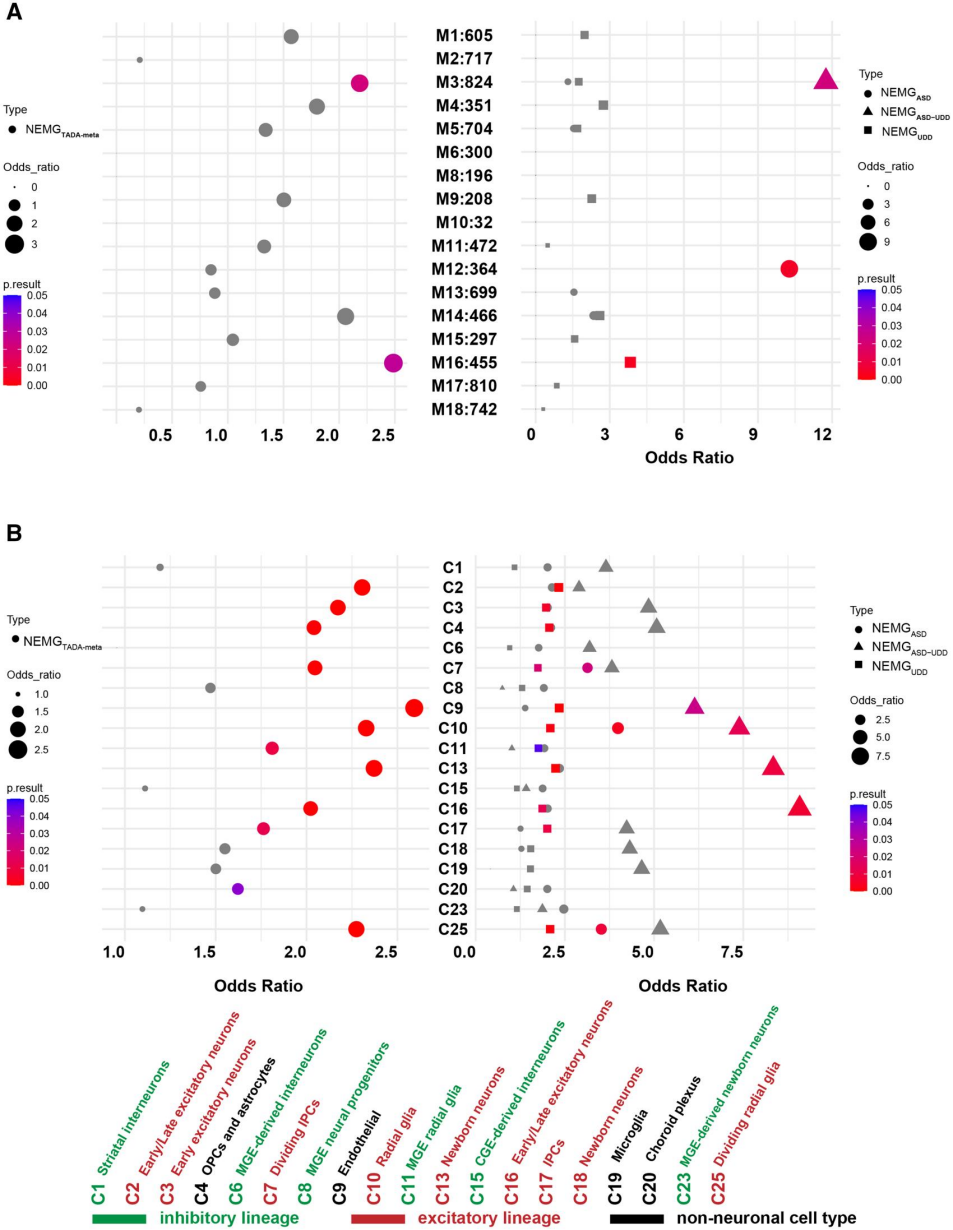
Expression pattern of disease-associated NEMGs. (**A**) Module-level enrichment of NEMG_ASD_, NEMG_UDD_, NEMG_ASD–UDD_ and NEMG_TADA-meta_. All enrichment values for overrepresented lists with *P*<0.05 and OR>1 are shown to demonstrate enrichment trends. (**B**) RNA-seq data from the human forebrain across the prenatal development were analyzed concerning the enrichment of NEMG_ASD_, NEMG_UDD_, NEMG_ASD–UDD_ and NEMG_TADA-meta_. ASD, autism spectrum disorder; UDD, undiagnosed developmental disorder; NEMG, nuclear-encoded mitochondrial gene; RPKM, reads per kilobase million.

We evaluated whether gene sets associated with UDD–ASD risk converged on common biological processes. To do so, we used the previously published BrainSpan spatial-temporal transcriptome data of the human brain and identified 22 co-expression modules.^19^ ASD-associated genes were differentially enriched in five co-expression modules during the development process: M2, M3, M13, M16 and M17, as described in detail by Parikshak *et al*.^19^ Genes in M2 and M3 were co-expressed during human cortical development and exhibited significant enrichment of transcriptional and chromatin regulators and genes harboring rare *de novo* protein-truncating and missense variants. Genes with high connectivity in M13, M16 and M17 were expressed in early cortical development and were enriched for genes encoding synaptic proteins. Notably, ASD-associated genes in M12 were only enriched for M13, M16 and M17. Genes in M16 were more strongly associated with ASD risk genes.^19^ Subsequently, we assessed whether our identified 130 genes (NEMG_ASD_, NEMG_UDD_ and NEMG_TADA-meta_) were significantly overrepresented within each of the co-expression modules. NEMG_TADA-meta_ genes were most overrepresented in M16 (OR = 2.49, *P*=0.028) and M3 (OR = 2.18, *P*=0.022) (Supplementary Table S9 and Figure 2A). Separation of the 130 genes according to phenotypic effect (NEMG_ASD_, NEMG_UDD_ and NEMG_ASD–UDD_) revealed a significant enrichment of NEMG_UDD_ and NEMG_ASD_ genes in the M16 module (OR = 3.83, *P*=0.0035) (Supplementary Table S9 and Figure 2A) and M12 module (OR = 10.27, *P*=0.0046) (Supplementary Table S9 and Figure 2A), respectively. These data provided some evidence to support a slight difference between NEMG_UDD_ and NEMG_ASD_ genes. Significant enrichment was observed NEMG_ASD–UDD_ in M3 module (OR = 11.75, *P* = 0.022) (Supplementary Table S9 and Figure 2A). NEMG_UDD_ and NEMG_ASD_ genes were enriched in different co-expression networks and showed different temporal expression patterns. An unbiased and systematic GO enrichment analysis further indicated that NEMG_UDD_ and NEMG_ASD_ genes are involved in processes related to the mitochondrial membrane, mitochondrial matrix, small molecule catabolic process and mitochondrion organization, whereas NEMG_ASD_ genes contributed mainly to basic and general functions, such as brain development, monoatomic cation homeostasis, positive regulation of DNA metabolic process and ATP-dependent activity (top-ranked GO terms) (Supplementary Table S10).

Next, we used a Wilcoxon signed-rank test to assess the relative pre- and postnatal expression bias for the gene sets. NEMG_TADA-meta_, NEMG_ASD_ and NEMG_UDD_ genes were associated with fetal development of the cerebral cortex (*P*=4.19 × 10^−12^, 2.49 × 10^−17^ and 4.32 × 10^−8^, respectively) (Supplementary Figure S5).

### Disease-associated NEMGs are enriched in excitatory and inhibitory neurons

To investigate the cell types implicated in UDD–ASD, we conducted an additional analysis in which we combined single-cell transcriptome data with the expression profiles of the NEMG_TADA-meta_, NEMG_ASD–UDD_, NEMG_ASD_ and NEMG_UDD_ gene sets. The single-cell RNA-seq dataset comprised 19 clusters from the human cerebral cortex (ranging within 6–37-weeks post-conception; average, 16.3-week post-conception). NEMG_TADA-meta_ (1.62 ≤ OR≤2.59; 2.01 × 10^−7^≤*P*_ _≤4.00 × 10^−2^) and NEMG_UDD_ (1.74 ≤ OR≤2.34; 1.53 × 10^−4^ ≤*P*_ _≤4.67 × 10^−2^) genes exhibit a heightened presence in maturing and mature neurons of excitatory and inhibitory lineages and non-neuronal cell types (endothelial cells, oligodendrocyte progenitor cells and astrocytes) (Supplementary Table S11 and Figure 2B). The major excitatory neuronal lineages, including dividing intermediate progenitor cells (C7), radial glia (C10) and dividing radial glia (C25), expressed mainly NEMG_ASD_ genes (3.13 ≤ OR≤3.99; 3.10 × 10^−3^≤*P*_ _≤2.21 × 10^−2^) (Supplementary Table S11 and Figure 2B). NEMG_ASD–UDD_ is enriched in excitatory neuronal lineages (C10 and C25) and endothelial cells (C9) (6.01 ≤ OR≤8.74; 1.31 × 10^−2^≤*P*_ _≤3.75 × 10^−2^) (Supplementary Table S11 and Figure 2B). These results point to the cellular location of UDD–ASD pathology and encourage future investigation to take the relationship of mitochondria to these cell types into account.

## Discussion

A critical challenge in UDD–ASD research includes the identification of disease-causing genes and a better understanding of their underlying molecular mechanisms. NEMGs act on mitochondrial function and affect gene expression and physiological regulation in the nucleus. Therefore, establishing abnormal regulation of NEMG as a source of molecular dysfunction contributing to UDD–ASD and further identifying specific pathogenic targets are key steps.

By examining NEMGs harboring DNVs, we found significant enrichment of PTVs and Dmis in patients with UDD–ASD relative to controls, with ascertainment differentials of 0.39% and 1.1% per affected child, respectively. This is a conservative estimate of the role of DNVs because we have not yet ascertained copy number variants or variants in non-coding regions.

Some of the top 20 NEMGs identified in this study exhibit concurrence with previously published findings. *DNMT3A* encodes a DNA methyltransferase and localizes in mitochondria to maintain CpH methylation in neurons *in vivo*^24^ and support the occurrence of mtDNA methylation. Knockout of *DNMT3A* disturbs regional mtDNA methylation patterns and alters the expression of mitochondrial genes (*MT-ND2* and *MT-CO1*), and the oxygen respiration process.^25^ Although DNMT3A is not specifically targeted to the mitochondria, it is identified in this organelle, indicating that it is a likely epigenetic mechanism for mtDNA methylation. SHP2, a non-receptor protein tyrosine phosphatase encoded by the *PTPN11* gene in humans, is predominantly localized within the mitochondria intercristae/intermembrane space, while also present in the cytoplasm and nucleus.^26^ Gain-of-function mutations in *PTPN11* have been implicated in neurodevelopmental processes related to human brain function, memory formation and attention regulation.^27^ A mutation in the X-linked pyruvate dehydrogenase (PDH) alpha subunit gene (*PDHA1*) results in a deficiency of the majority PDH complex, which affects mitochondrial function and may contribute to brain dysfunction in autism.^28^^,^^29^ A study has implicated *DNM1L* variants in causing impaired mitochondrial function and a reduced response to stress in patients with severe neurological dysfunction.^30^ Hypusinated eIF5A promotes the efficient expression of a subset of mitochondrial proteins involved in the TCA cycle and OXPHOS. *De novo* heterozygous variants in *EIF5A* result in a disorder characterized by varying combinations of developmental delay, microcephaly, micrognathia and dysmorphism.^31^ Clinical manifestations of developmental disorders are caused by mutations or disruptions in the *POLG* gene, which is responsible for encoding the catalytic subunit of mitochondrial DNA polymerase gamma.^32^ Although the association between the genes in Table 2 and mitochondrial functions in ASD and UDD remains largely unexplored, these few examples highlight the enormous potential of these molecules as messengers in the crosstalk between the nucleus and the mitochondria.

Although genes we identified to harbor dDNVs herein showed statistical significance in the TADA-*denovo* model, these represent only potential candidates for future investigations, as opposed to true disease-causing genes. The two hub NEMGs (*ATP5F1A* and *ATP5F1B*) (Supplementary Table S9) both encode a subunit of the mitochondrial ATP synthase, which is responsible for >90% of the cellular ATP synthesis. Among heterozygous *ATP5F1A*-related mitochondrial disorders is reversible neonatal mitochondrial disorder (OMIM #500009), which is caused by mutations in the mitochondrial gene *MT-TE.*^33^*ATP5F1B* was previously identified in a study that explored ultra-rare inherited variants implicated in ASD.^18^ Moreover, we predicted *MT-ATP6* to be a highly probable functional partner of *ATP5F1B*. Further studies are needed to understand the role of the nucleus–mitochondria-crosstalk signaling for UDD–ASD.

Lastly, we performed a detailed analysis of NEMGs that predominantly conferred a risk for UDD–ASD, as biological function analyses revealed a clear difference between the two sets of genes. The small effect of *de novo* mutations in NEMG_ASD_ relative to NEMG_UDD_ may indicate that individuals carrying dDNVs in NEMGs show a broad phenotype of UDD–ASD, without all core symptoms of ASD. This distinction has important implications for researchers because it suggests that determining the impact of these NEMGs could contribute to the classification of subtypes of UDD–ASD and tailor new methods for intervening in patients with this specific mitochondrial dysfunction. Larger cohorts will be needed in the future to validate our results and explain the differences in functional mechanisms through wet experiments. Single-cell gene expression data from the developing human cortex further showed that endothelial cells, as well as mature neurons from both excitatory and inhibitory lineages, may be implicated in ASD risk. In particular, a significant enrichment of NEMG_TADA-meta,_ NEMG_UDD_ and NEMG_ASD–UDD_ was observed in brain endothelial cells. Segarra *et al*.^34^ reported that communication between endothelial and glial cells is responsible for coordinating neuronal migration and the blood–brain barrier establishment in mice. Moreover, brain endothelial cells can directly regulate the synaptic plasticity of hippocampal neurons by secreting Sema3G and participating in brain cognition.^35^ However, although the endothelium plays an essential role in brain function, it remains unclear how signals from NEMG_UDD_ in brain endothelial cells orchestrate communication among vessels, glial cells and neurons, and how specific changes in the molecular characteristics of endothelial cells affect processes, such as central nervous system vascularization, extracellular matrix composition, neuroglial cytoarchitecture and blood–brain barrier development.

There are several limitations of this study. Given the considerably smaller size of the control sample, although we were able to successfully replicate the same outcomes in the burden analysis step using a downsized permutation test, future investigations must employ larger control sample sizes to substantiate our findings. We acknowledge that the number of NEMGs from databases used here may still need to be accurately and comprehensively defined, but they encompass a wealth of gene information related to sub-mitochondrial localization or ontologically linked to each category or pathway. Despite expanding the set of known associations between NEMGs and UDD–ASD, further studies are needed to understand the role of the nucleus–mitochondria-crosstalk signaling for UDD–ASD. Our analysis relied on bioinformatics methodologies to prioritize potential disease-associated genes and cannot definitively establish causality. While we chose to focus on 130 disease-associated NEMGs (FDR<0.2), some of these may not be truly causal, whereas other (FDR>0.2) associations may be causal.

In conclusion, based on dDNVs in NEMGs, we prioritized 130 UDD–ASD-associated NEMGs that showed the potential to shed light on the role of mitochondria in UDD–ASD pathogenesis. NEMGs involved in mitochondrial metabolism are candidates’ worthy of further research concerning UDD–ASD. If NEMG mutation in UDD–ASD creates an aberrant neuronal ‘wiring diagram’ in fetal brain development, this may be difficult to repair. However, if NEMG mutation in a UDD–ASD subtype results in mild mitochondrial inhibition, NEMGs may represent a viable option and promising therapeutic intervention target for metabolic therapies in some patients.

## Supplementary Material

hcad249_Supplementary_Data

## References

[1] Thapar A , Cooper M, Rutter M. Neurodevelopmental disorders. Lancet Psychiatry 2017; 4:339–46. 27979720 10.1016/S2215-0366(16)30376-5

[2] Wallace DC. Mitochondrial genetic medicine. Nat Genet 2018; 50:1642–9. 30374071 10.1038/s41588-018-0264-z

[3] Wang Y , Guo X, Hong X, Wang G, Pearson C, Zuckerman B, et al Association of mitochondrial DNA content, heteroplasmies and inter-generational transmission with autism. Nat Commun 2022; 13:3790. 35778412 10.1038/s41467-022-30805-7 PMC9249801

[4] Glessner JT , Wang K, Cai G, Korvatska O, Kim CE, Wood S, et al Autism genome-wide copy number variation reveals ubiquitin and neuronal genes. Nature 2009; 459:569–73. 19404257 10.1038/nature07953 PMC2925224

[5] Brown CO , Uy J, Murtaza N, Rosa E, Alfonso A, Xing S, et al Disruption of the autism-associated gene SCN2A alters synaptic development and neuronal signaling in patient iPSC-glutamatergic neurons. bioRxiv, 2021, 2021.09.14.460368. 10.3389/fncel.2023.1239069 PMC10824931 38293651

[6] Diodato D , Melchionda L, Haack TB, Dallabona C, Baruffini E, Donnini C, et al VARS2 and TARS2 mutations in patients with mitochondrial encephalomyopathies. Hum Mutat 2014; 35:983–9. 24827421 10.1002/humu.22590 PMC4140549

[7] Replogle JM , Saunders RA, Pogson AN, Hussmann JA, Lenail A, Guna A, et al Mapping information-rich genotype-phenotype landscapes with genome-scale perturb-seq. Cell 2022; 185:2559–75.e28. 35688146 10.1016/j.cell.2022.05.013 PMC9380471

[8] Rossignol DA , Frye RE. Mitochondrial dysfunction in autism spectrum disorders: a systematic review and meta-analysis. Mol Psychiatry 2012; 17:290–314. 21263444 10.1038/mp.2010.136 PMC3285768

[9] Satterstrom FK , Kosmicki JA, Wang J, Breen MS, De Rubeis S, An JY, et al Large-scale exome sequencing study implicates both developmental and functional changes in the neurobiology ofautism. Cell 2020; 180:568–84.e23. 31981491 10.1016/j.cell.2019.12.036 PMC7250485

[10] Rath S , Sharma R, Gupta R, Ast T, Chan C, Durham TJ, et al MitoCarta3.0: an updated mitochondrial proteome now with Sub-organelle localization and pathway annotations. Nucleic Acids Res 2021; 49:D1541–7. 33174596 10.1093/nar/gkaa1011 PMC7778944

[11] Smith AC , Robinson AJ. MitoMiner v4.0: an updated database of mitochondrial localization evidence, phenotypes and diseases. Nucleic Acids Res 2019; 47:D1225–8. 30398659 10.1093/nar/gky1072 PMC6323904

[12] Wang K , Li M, Hakonarson H. ANNOVAR: functional annotation of genetic variants from high-throughput sequencing data. Nucleic Acids Res 2010; 38:e164. 20601685 10.1093/nar/gkq603 PMC2938201

[13] Ware JS , Samocha KE, Homsy J, Daly MJ. Interpreting de novo variation in human disease using denovolyzeR. Curr Protoc Hum Genet 2015; 87:7.25.1–15. 10.1002/0471142905.hg0725s87 PMC4606471 26439716

[14] Iossifov I , O'Roak BJ, Sanders SJ, Ronemus M, Krumm N, Levy D, et al The contribution of de novo coding mutations to autism spectrum disorder. Nature 2014; 515:216–21. 25363768 10.1038/nature13908 PMC4313871

[15] He X , Sanders SJ, Liu L, De Rubeis S, Lim ET, Sutcliffe JS, et al Integrated model of de novo and inherited genetic variants yields greater power to identify risk genes. PLoS Genet 2013; 9:e1003671. 23966865 10.1371/journal.pgen.1003671 PMC3744441

[16] Karczewski KJ , Francioli LC, Tiao G, Cummings BB, Alfoldi J, Wang Q, et al; Genome Aggregation Database Consortium. The mutational constraint spectrum quantified from variation in 141,456 humans. Nature 2020; 581:434–43. 32461654 10.1038/s41586-020-2308-7 PMC7334197

[17] Kaplanis J , Samocha KE, Wiel L, Zhang Z, Arvai KJ, Eberhardt RY, et al; Deciphering Developmental Disorders Study. Evidence for 28 genetic disorders discovered by combining healthcare and research data. Nature 2020; 586:757–62. 33057194 10.1038/s41586-020-2832-5 PMC7116826

[18] Wilfert AB , Turner TN, Murali SC, Hsieh P, Sulovari A, Wang T, et al; SPARK Consortium. Recent ultra-rare inherited variants implicate new autism candidate risk genes. Nat Genet 2021; 53:1125–34. 34312540 10.1038/s41588-021-00899-8 PMC8459613

[19] Parikshak NN , Luo R, Zhang A, Won H, Lowe JK, Chandran V, et al Integrative functional genomic analyses implicate specific molecular pathways and circuits in autism. Cell 2013; 155:1008–21. 24267887 10.1016/j.cell.2013.10.031 PMC3934107

[20] Zhou Y , Zhou B, Pache L, Chang M, Khodabakhshi AH, Tanaseichuk O, et al Metascape provides a biologist-oriented resource for the analysis of systems-level datasets. Nat Commun 2019; 10:1523. 30944313 10.1038/s41467-019-09234-6 PMC6447622

[21] Nowakowski TJ , Bhaduri A, Pollen AA, Alvarado B, Mostajo-Radji MA, Di Lullo E, et al Spatiotemporal gene expression trajectories reveal developmental hierarchies of the human cortex. Science 2017; 358:1318–23. 29217575 10.1126/science.aap8809 PMC5991609

[22] Luo T , Li K, Ling Z, Zhao G, Li B, Wang Z, et al De novo mutations in folate-related genes associated with common developmental disorders. Comput Struct Biotechnol J 2021; 19:1414–22. 33777337 10.1016/j.csbj.2021.02.011 PMC7966843

[23] Bamshad MJ , Nickerson DA, Chong JX. Mendelian gene discovery: fast and furious with no end in sight. Am J Hum Genet 2019; 105:448–55. 31491408 10.1016/j.ajhg.2019.07.011 PMC6731362

[24] Guo JU , Su Y, Shin JH, Shin J, Li H, Xie B, et al Distribution, recognition and regulation of non-CpG methylation in the adult mammalian brain. Nat Neurosci 2014; 17:215–22. 24362762 10.1038/nn.3607 PMC3970219

[25] Dou X , Boyd-Kirkup JD, McDermott J, Zhang X, Li F, Rong B, et al The strand-biased mitochondrial DNA methylome and its regulation by DNMT3A. Genome Res 2019; 29:1622–34. 31537639 10.1101/gr.234021.117 PMC6771398

[26] Arachiche A , Augereau O, Decossas M, Pertuiset C, Gontier E, Letellier T, et al Localization of PTP-1B, SHP-2, and Src exclusively in rat brain mitochondria and functional consequences. J Biol Chem 2008; 283:24406–11. 18583343 10.1074/jbc.M709217200 PMC3259839

[27] Xu D , Zheng H, Yu WM, Qu CK. Activating mutations in protein tyrosine phosphatase Ptpn11 (Shp2) enhance reactive oxygen species production that contributes to myeloproliferative disorder. PLoS One 2013; 8:e63152. 23675459 10.1371/journal.pone.0063152 PMC3651249

[28] Giulivi C , Zhang YF, Omanska-Klusek A, Ross-Inta C, Wong S, Hertz-Picciotto I, et al Mitochondrial dysfunction in autism. JAMA 2010; 304:2389–96. 21119085 10.1001/jama.2010.1706 PMC3915058

[29] Lissens W , De Meirleir L, Seneca S, Liebaers I, Brown GK, Brown RM, et al Mutations in the X-linked pyruvate dehydrogenase (E1) alpha subunit gene (PDHA1) in patients with a pyruvate dehydrogenase complex deficiency. Hum Mutat 2000; 15:209–19. 10679936 10.1002/(SICI)1098-1004(200003)15:3<209::AID-HUMU1>3.0.CO;2-K

[30] Hogarth KA , Costford SR, Yoon G, Sondheimer N, Maynes JT. DNM1L variant alters baseline mitochondrial function and response to stress in a patient with severe neurological dysfunction. Biochem Genet 2018; 56:56–77. 29110115 10.1007/s10528-017-9829-2

[31] Faundes V , Jennings MD, Crilly S, Legraie S, Withers SE, Cuvertino S, et al Impaired eIF5A function causes a Mendelian disorder that is partially rescued in model systems by spermidine. Nat Commun 2021; 12:833. 33547280 10.1038/s41467-021-21053-2 PMC7864902

[32] Uusimaa J , Gowda V, McShane A, Smith C, Evans J, Shrier A, et al Prospective study of POLG mutations presenting in children with intractable epilepsy: prevalence and clinical features. Epilepsia 2013; 54:1002–11. 23448099 10.1111/epi.12115 PMC3757309

[33] Lines MA , Cuillerier A, Chakraborty P, Naas T, Duque Lasio ML, Michaud J, et al A recurrent de novo ATP5F1A substitution associated with neonatal complex V deficiency. Eur J Hum Genet 2021; 29:1719–24. 34483339 10.1038/s41431-021-00956-0 PMC8560863

[34] Segarra M , Aburto MR, Cop F, Llao-Cid C, Hartl R, Damm M, et al Endothelial Dab1 signaling orchestrates neuro-glia-vessel communication in the central nervous system. Science 2018; 361:eaao2861. 30139844 10.1126/science.aao2861

[35] Tan C , Lu NN, Wang CK, Chen DY, Sun NH, Lyu H, et al Endothelium-derived semaphorin 3G regulates hippocampal synaptic structure and plasticity via neuropilin-2/PlexinA4. Neuron 2019; 101:920–37.e13. 30685224 10.1016/j.neuron.2018.12.036

