# Supplementary material for: Association between de novo variants of nuclear-encoded mitochondrial-related genes and undiagnosed developmental disorder and autism
Source: QJM. 2023 Nov 1;117(4):269–76. doi: 10.1093/qjmed/hcad249 (PMC11014680; doi:10.1093/qjmed/hcad249)
Supplement: hcad249_Supplementary_Data [file hcad249_supplementary_data.zip › hcad249_Supplementary_Data/Supplementary figure.docx]

**Supplementary Figures**


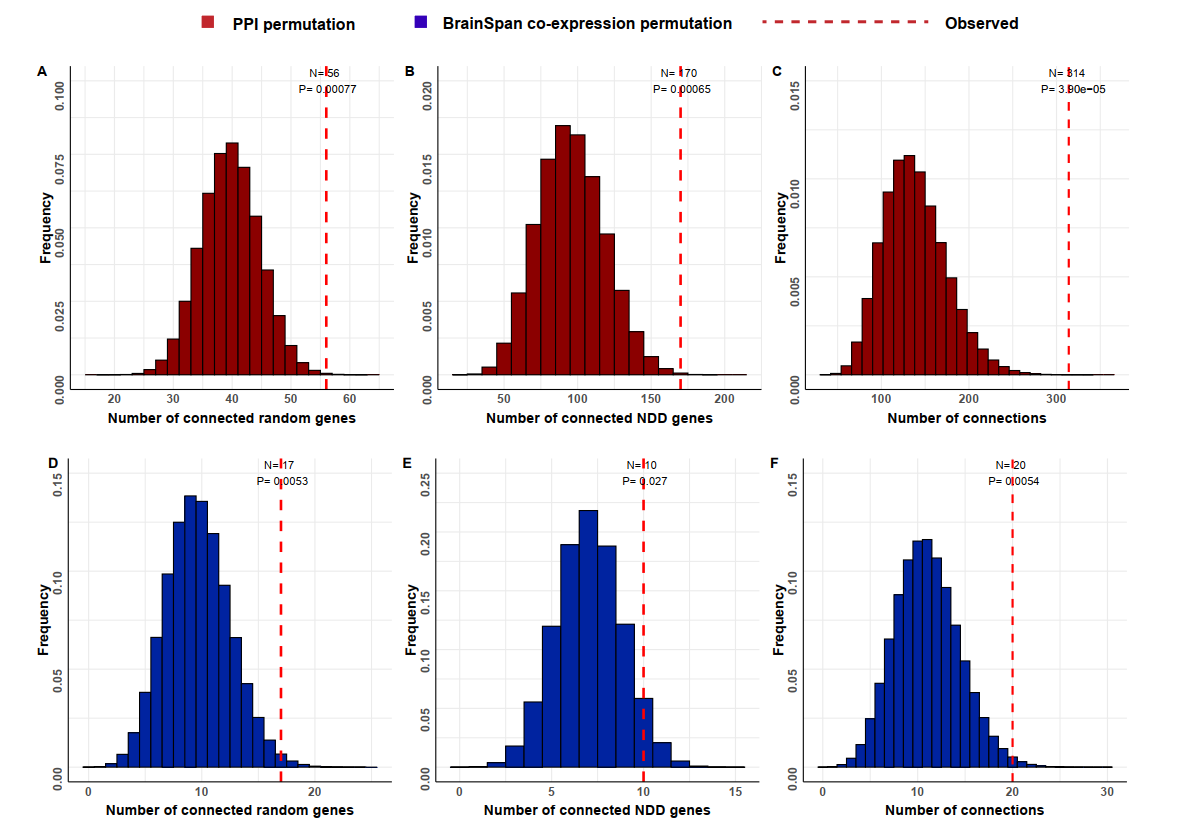


**Supplementary Figure 1.** Permutation figures of connections between 130 disease-associated NEMGs and the unshared 627 NDD risk genes based on PPI and BrainSpan co-expression data compared to 1,000,000 random selections. Empirical p-values calculated from permutation tests.


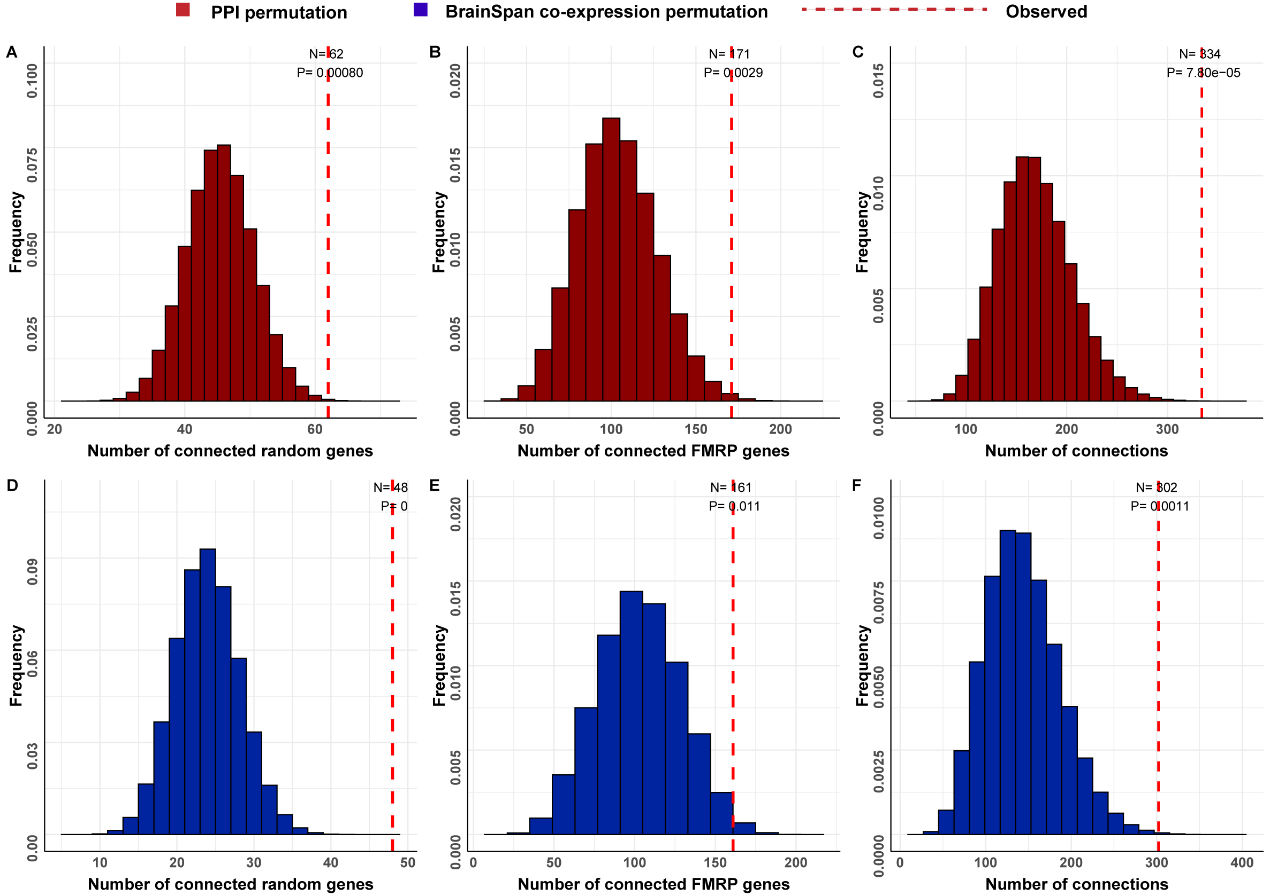


**Supplementary Figure 2.** Permutation figures of connections between 130 disease-associated NEMGs and the unshared 842 genes encoded Fragile-X mental retardation protein (FMRP)[1] based on PPI and BrainSpan co-expression data compared to 1,000,000 random selections. Empirical p-values calculated from permutation tests.


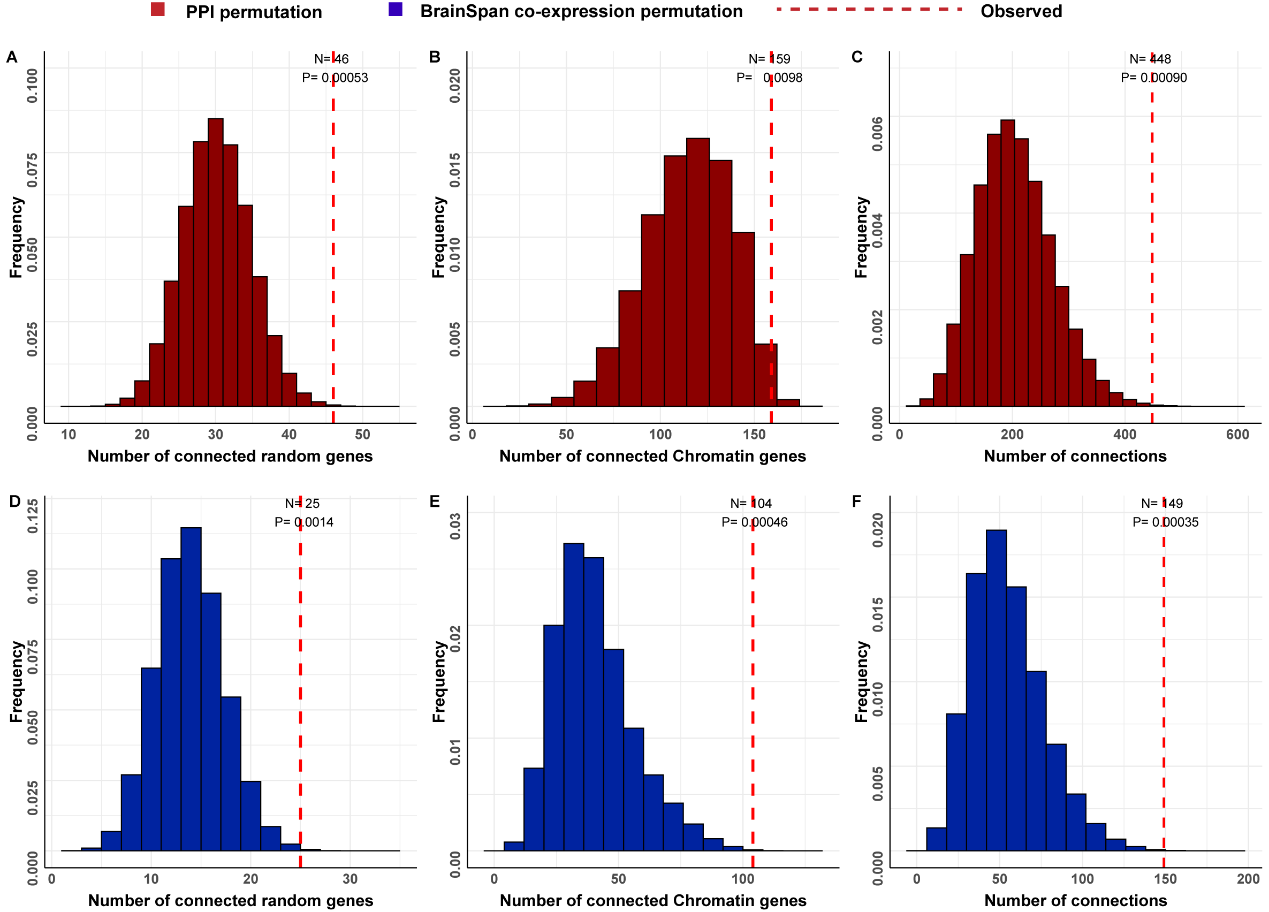


**Supplementary Figure 3.** Permutation figures of connections between 130 disease-associated NEMGs and the unshared 408 chromatin genes[2] based on PPI and BrainSpan co-expression data compared to 1,000,000 random selections. Empirical p-values calculated from permutation tests.


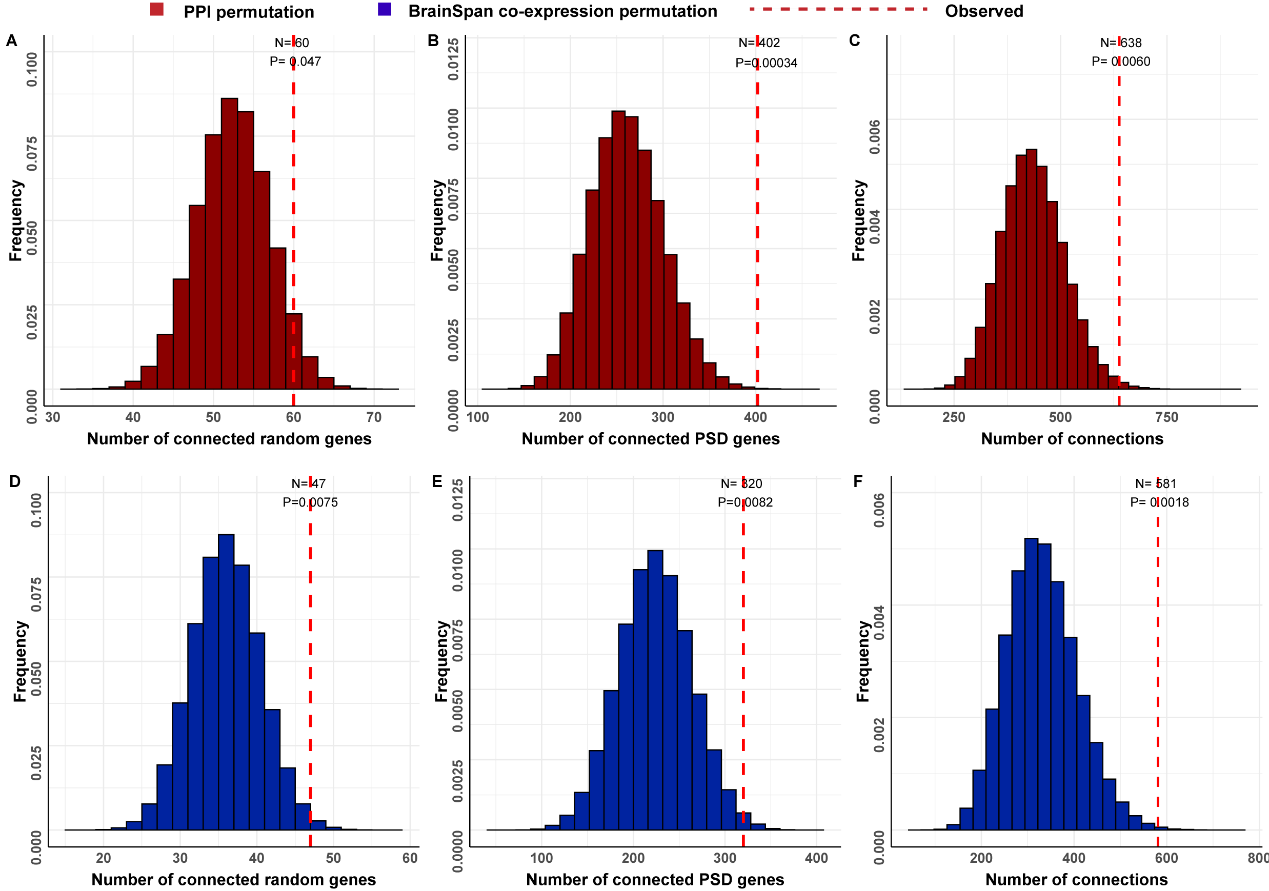
**Supplementary Figure 4.** Permutation figures of connections between 130 disease-associated NEMGs and the unshared 1,422 genes encoded postsynaptic density proteins (PSD)[3] based on PPI and BrainSpan co-expression data compared to 1,000,000 random selections. Empirical p-values calculated from permutation tests.


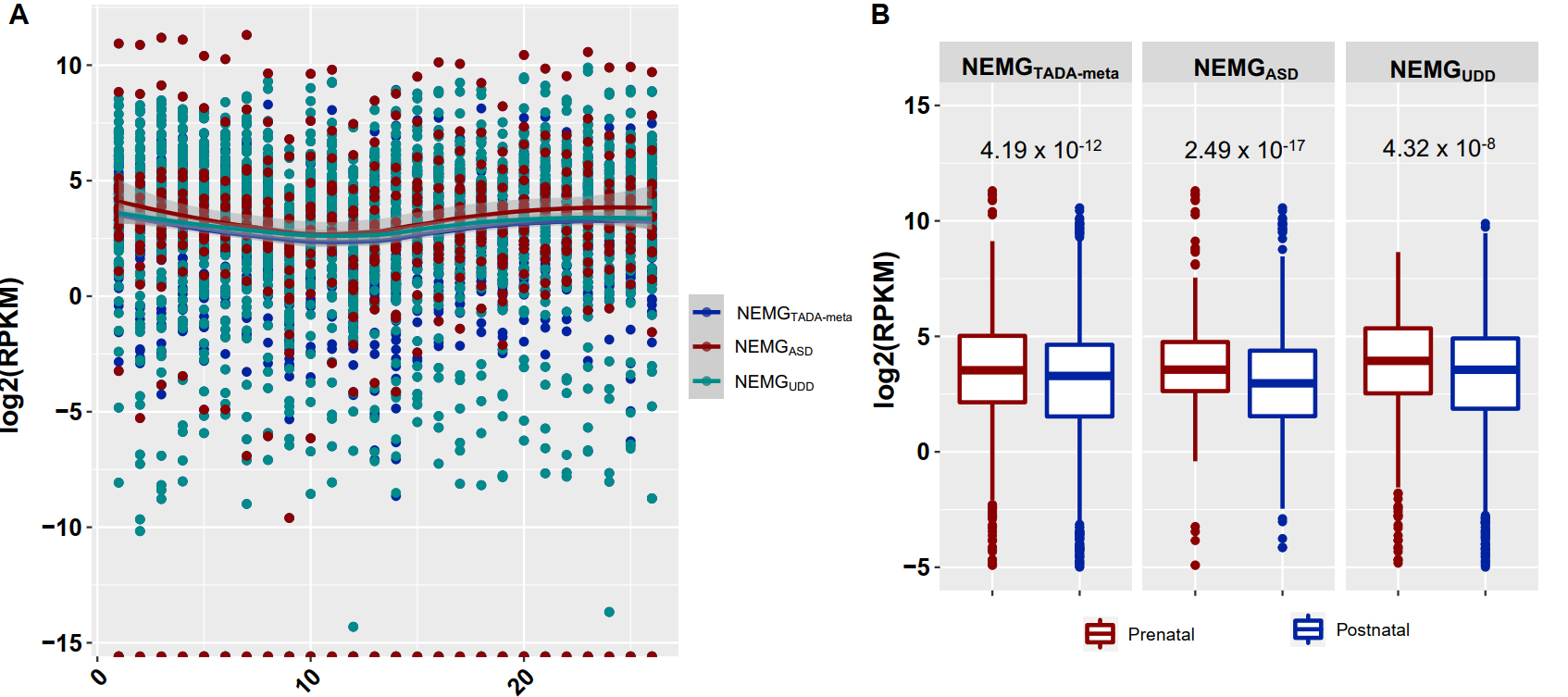


**Supplementary Figure 5.** Expression patterns of NEMG_ASD_, NEMG_UDD_, and NEMG_ASD-UDD_ gene sets across developmental periods of the brain cortex. A Wilcoxon signed-rank test was performed to compare the prenatal and postnatal expression of the gene sets in the BrainSpan data. The y-axis represents the log2-transformed RPKM of genes. Bonferroni adjusted p-values calculated by a Wilcoxon signed-rank test are shown.

[1] Darnell JC, Van Driesche SJ, Zhang C, Hung KY, Mele A, Fraser CE, et al. FMRP stalls ribosomal translocation on mRNAs linked to synaptic function and autism. Cell. 2011;146(2):247-61.

[2] Iossifov I, O'Roak BJ, Sanders SJ, Ronemus M, Krumm N, Levy D, et al. The contribution of de novo coding mutations to autism spectrum disorder. Nature. 2014;515(7526):216-21.

[3] Bayes A, van de Lagemaat LN, Collins MO, Croning MD, Whittle IR, Choudhary JS, et al. Characterization of the proteome, diseases and evolution of the human postsynaptic density. Nat Neurosci. 2011;14(1):19-21.
